# Tree Rings Mercury Controlled by Atmospheric Gaseous Elemental Mercury and Tree Physiology

**DOI:** 10.1021/acs.est.4c05662

**Published:** 2024-09-09

**Authors:** Haijun Peng, Xiangwen Zhang, Kevin Bishop, John Marshall, Mats B. Nilsson, Chuxian Li, Erik Björn, Wei Zhu

**Affiliations:** † Department of Forest Ecology and Management, Swedish University of Agricultural Sciences, Umeå SE-90183, Sweden; ‡ School of Resources & Environment, Nanchang University, Nanchang 330031, China; § Department of Aquatic Sciences and Assessment, Swedish University of Agricultural Sciences, Uppsala SE-75651, Sweden; ∥ Institute of Geography and Oeschger Center for Climate Change Research, University of Bern, Bern 3012, Switzerland; ⊥ Department of Chemistry, Umeå University, Umeå SE-901 87, Sweden

**Keywords:** tree rings, natural archive, atmospheric Hg, tree physiology, xylem nitrogen transport, radial translocation, dendrochemistry

## Abstract

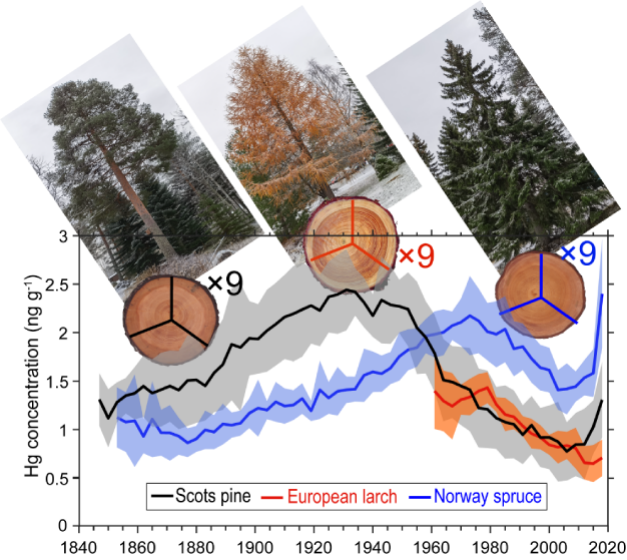

Tree rings are an emerging atmospheric mercury (Hg) archive. Questions have arisen, though, regarding their mechanistic controls and reliability. Here, we report contrasting tree-ring Hg records in three collocated conifer species: Norway spruce (*Picea abies*), Scots pine (*Pinus sylvestris*), and European larch (*Larix decidua*), which are from a remote boreal forest. Centennial atmospheric Hg trends at the site, derived from varved lake sediments, peats, and atmospheric monitoring, indicated a steady rise from the 1800s, peaking in the 1970s, and then declining. Prior to ca. 2005, larch and spruce tree rings reproduced the peak in the atmospheric Hg trend, while pine tree rings peaked in the 1930s, likely due to the prolonged sapwood period and ambiguity in the heartwood–sapwood boundary of pine. Since ca. 2005, tree rings from all species showed increasing Hg concentrations in the physiologically active outer rings despite declining atmospheric Hg concentrations. The good agreement between Hg and nitrogen concentrations in active tree-ring cells indicates a similar transport mechanism and cautions against their applicability as atmospheric Hg archives. Our results suggest that tree-ring Hg records are controlled by atmospheric Hg and tree physiology. We provide recommendations for using tree-ring Hg archives that take tree physiology into account.

## Abstract 2

This study demonstrates that tree-ring Hg records are jointly controlled by atmospheric Hg concentrations and tree physiology, with important implications for using tree rings to reconstruct past atmospheric Hg.

## 1 Introduction

Mercury (Hg) is a highly toxic pollutant of global concern.^1^ Owing to its long atmospheric residence time (∼0.5–1 year), Hg is dispersed globally via long-distance transport of elemental Hg [Hg(0)].^2,3^ Hg deposited into aquatic ecosystems can be methylated by microbes, forming methylmercury (MeHg). This is a neurotoxin that can bioaccumulate up to 10^6^ times in food webs, and it is a threat to people and wildlife. For humans, MeHg exposure is associated with long-term neurocognitive deficits in children and cardiovascular impairments in adults.^4^ The intergovernmental treaty Minamata Convention on Mercury, initiated by the United Nations, has united more than 140 countries since 2013 to reduce Hg emissions and protect humans and wildlife from Hg exposure. Atmospheric Hg concentration can be influenced by many emission sources (e.g., volcanic activities, artisanal gold mining, coal combustion, and nonferrous metals production), as well as re-emission (e.g., evasion from land and water surfaces) and deposition (e.g., vegetation uptake) processes.^2,5−7^ This can result in large variability of Hg concentration in the local, regional, and global atmosphere. Knowledge of historical atmospheric Hg concentration is essential to understanding Hg cycles in the biosphere and to evaluate the effectiveness of the Minamata Convention.^8^ In situ atmospheric Hg measurements began in the 1970s, with long-term monitoring starting in the 1990s, at a limited number of stations.^9^ Due to the lack of long-term instrumental Hg measurement data, studies on environmental archives, such as lake sediments,^10−12^ peat deposits,^13−15^ and ice cores^16−19^ have been extensively used to reconstruct atmospheric Hg depositions and concentrations. Compared to other natural archives, tree rings are emerging as an archive of atmospheric Hg for several reasons: (1) trees are broadly distributed globally, (2) trees allow precise and high resolution dating at low cost, and (3) trees record atmospheric Hg concentration rather than Hg deposition flux.^20−24^

The fundamental principle of Hg dendrochemistry is that the accumulation of Hg in a tree-ring originates from assimilation of atmospheric Hg(0), allowing the establishment of a linear relationship between the concentrations of Hg recorded in the annual growing tree-ring and those in the atmosphere.^23^ Recent studies have observed that a number of conifer species (e.g., European larch, Huon pine, white spruce, Norway spruce, and Masson pine) exhibit Hg concentration patterns in tree rings that align with documented histories of Hg pollution.^22,25−28^ However, no quantitative relationship between the Hg concentrations in tree rings and atmospheric Hg concentrations at those contaminated sites has been established. Tree rings from some broadleaf tree species have proven unsuitable for archiving atmospheric Hg trends,^21,29^ even at contaminated sites.^29,30^ While at noncontaminated sites, only a few conifer species, such as European larch^31^ and white spruce,^32^ have been found to record regional atmospheric Hg concentration history that are similar to other archives or inventories. But even for these more suitable tree species, questions remain, both regarding the quantitative timing of trends, and especially, the patterns in the outermost tree rings.^24,27^ Recent reviews therefore concluded that while Hg concentrations in tree rings may be related to atmospheric Hg, they are also influenced by other environmental (e.g., meteorological, geographical, and geomorphological conditions) as well as physiological factors (e.g., tree species, tree ages, radial translocations).^23,24^

Trees assimilate Hg mainly via foliar uptake (both stomatal and nonstomatal pathways) from the atmosphere, which is then transported to the outermost tree ring through downward phloem transport.^20,35−37^ From there, the Hg may or may not be transported radially across the sapwood via the living cells of the parenchyma.^33^ Comparison of tree-ring Hg records among various tree species and between tree rings and ice cores suggests that radial translocation may account for inconsistencies in Hg records within tree rings of some species.^34,35^ This underscores a crucial knowledge gap regarding the mechanistic controls of Hg accumulation in tree rings. As long as that knowledge gap exists, it compromises the foundation for utilizing tree rings in reconstructing atmospheric Hg concentrations.^23,24^ The key challenge for resolving this gap lies in deciphering the factors and processes that influence Hg assimilation and mobilization in tree rings and then assessing how these processes may affect the reliability of employing tree rings as archives for atmospheric Hg(0).

We propose that the assimilation and mobilization of Hg in tree rings are closely linked to processes that transport nutrients across the cytoplasm of living cells. Because nitrogen concentrations reflect the living cytoplasm in the parenchyma cells in the xylem, we used nitrogen concentrations to assess the importance of this physiological pathway. In this study, we determined high-resolution Hg and nitrogen concentrations in tree rings from collocated Norway spruce (*Picea abies*), Scots pine (*Pinus sylvestris*), and European larch (*Larix decidua*) trees in northern Sweden. We assess the reliability of utilizing tree rings from these three conifer species as atmospheric Hg(0) archives by comparing the Hg records in tree rings with those from adjacent peat and lake sediment archives as well as long-term atmospheric Hg(0) monitoring data. We also discuss the lifecycle processes of wood cells that regulate Hg assimilation, mobilization, and stabilization in tree rings. The paper concludes with recommendations for how to use tree rings to reconstruct atmospheric Hg(0) concentrations.

## 2 Materials and Methods

### 2.1 Site Description

**Figure 1:**
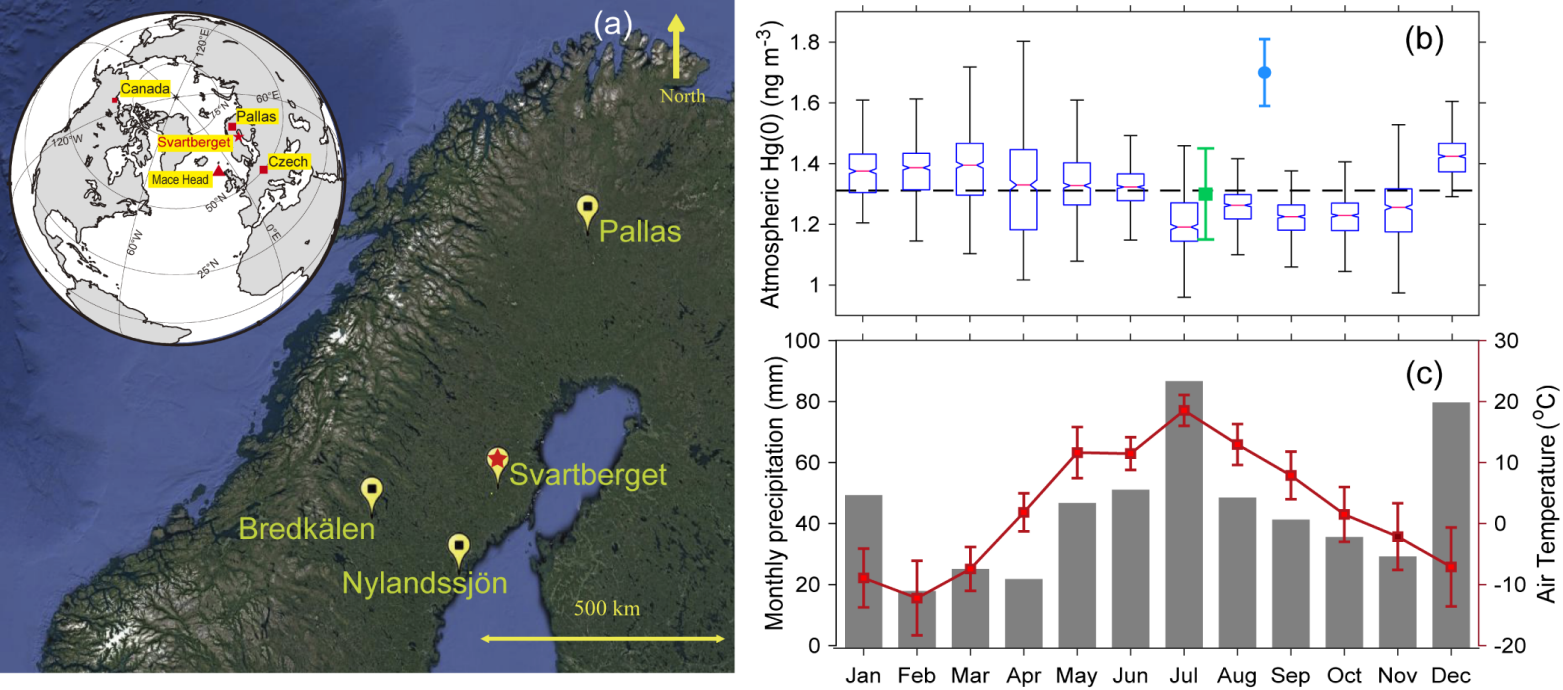
(a) Map showing the location of the tree ring sampling sites in this study (Svartberget, northern Sweden) and cited literature from other remote sites in North America (Canada)^32^ and Central Europe (Czech Republic).^31^ Nylandssjön is a remote boreal lake in which Hg was recorded in the varved sediments.^46^ Pallas, Bredkälen, and Mace Head are regional background sites that have long-term atmospheric gaseous Hg concentration measurements. (b) Box plot of the atmospheric Hg(0) concentration measured at Svartberget in 2018 (dashed horizontal line represents the annual mean value), with blue circle and green square error bars showing the Hg(0) values measured in August 2009^61^ and July 2014,^62^ respectively. (c) Monthly precipitation (gray bars) and mean air temperature (red error bars) at Svartberget in 2018.

The Vindeln Experimental Forest (Svartberget, 64°14′N, 19°46′E, Figure 1), located approximately 50 km northwest of Umeå in northern Sweden, is a typical remote boreal forest.^36,37^ The area is characterized by glacial till derived from gneissic and granitic bedrock, where soils are dominated by typical upland podzols. Soil pH was acidic in organic soils (∼4.0) and moderately acidic in mineral soils (∼4.5–5.5). Soil total Hg concentrations in the podzols ranged from 100 to 300 ng g^–1^ in organic soils and 5–50 ng g^–1^ in mineral soils, consistent with previously reported values at remote boreal forests in northern Sweden^38^ and Canada.^39^ The site has a cold–humid climate, with July being the hottest month and February being the coldest (Figure 1c). Thirty-year (1986–2015) mean annual precipitation and air temperature were 619 mm and 2.1 °C.^40^ The growing season is approximately six months, usually beginning in early May and ending in late October, with snow cover for the remainder of the year. The dominant tree species in the forest consists of Scots pine (*Pinus sylvestris*, ∼63%), Norway spruce (*Picea abies*, ∼26%), and birch (*Betula* spp.).^36^ Tree rings of Norway spruce and Scots pine trees were collected in the center of the Svartberget forest (64°15′13″N, 19°47′42″E) in July 2020, while samples from European larch (*Larix decidua*) were collected approximately 15 km away from the Scots pine and Norway spruce trees (64°14′58″N, 19°48′49″E) in August 2020.

### 2.2 Tree Ring Collection, Processing, and Chemical Analysis

For each of the three species, nine living trees with no injuries were selected for tree-ring sampling. To account for potential variations around the tree bole, three cores were taken from each tree, evenly spaced ∼120° around the circumference, resulting in 81 tree-ring cores from 27 randomly selected trees for Hg concentration analysis.^20^ Similarly, a second set of 81 tree-ring cores with each core located just a few centimeters below the first drilling point was taken for heartwood–sapwood boundary characterization and total nitrogen (N) analysis. The tree-ring cores were taken at chest height (∼1.5 m above ground) using a 5.1 mm Haglöf increment borer (Haglöf Sweden AB, Långsele, Sweden). To avoid cross-contamination from the outer bark, the outer bark surrounding the sampling point (∼1.5 × 1.5 cm^2^) was removed prior to each drilling with a precleaned stainless-steel knife. The cores were placed in clean paper tree-core tubes for transportation and then stored at −18 °C within 6 h until further processing.^31^

Before cross-dating and slicing, the surface of the tree ring core was slightly cleaned by using a stainless-steel blade to avoid cross-contamination and to improve visualization for cross-dating. Tree-ring cores were then dated at least twice under a microscope to avoid miscounting and false rings.^28^ The cores were then sliced into 3-year segments using a stainless-steel blade under a microscope. The blade was cleaned with tissue paper and ethanol before use. The oldest sampled tree rings of Norway spruce, Scots pine, and European larch were dated to 1853, 1847, and 1961, respectively. The subsamples representing a single three-year period from the three cores (first set of tree cores) from the same tree were merged into one composite sample to increase representativeness and decrease variability in the Hg analysis. Each composite sample was placed in a clean paper bag and oven-dried at 60 °C for 72 h. For the second set of tree cores, one of the three cores from each tree was selected for the heartwood–sapwood boundary identification; the other two cores were dated, sliced, stored, and dried as above to prepare them for N analysis. The composite sample for N analysis was milled and homogenized with a ball mill (Retsch MM400).

Total Hg concentrations of tree ring samples were determined on a direct mercury analyzer (DMA-80 Tricell, Milestone, Sorisole, Italy). Each composite sample was measured once on the DMA-80. One reference material (pine needles, NIST 1575a) and one blank were measured after 10 tree-ring samples. The mean concentration and recovery of the reference materials were 38.6 ± 0.2 ng g^–1^ and 93.2% (±SD, *n* = 113), respectively, indicating a good recovery and high reproducibility of the Hg analyses. The Hg concentration representing a 3-year interval for each tree species was reported as the mean ± SD of nine individual trees.

The heartwood–sapwood boundary was identified under a microscope.^41−43^ Total nitrogen (N) concentration and stable N isotope ratios (δ^15^N) were determined on an elemental analyzer–isotope ratio mass spectrometer (EA-IRMS) system, which is composed of an elemental analyzer (Flash EA 2000, Thermo Fisher Scientific, Bremen, Germany) and an isotope ratio mass spectrometer (DeltaV, Thermo Fisher Scientific, Bremen, Germany). The method^44^ was tuned by increasing helium dilution to attenuate CO_2_ signal and extending residual CO_2_ flushing time for precise analysis of low N concentrations. A composite tree-ring sample was used for triplicate analysis, resulting in a variability of 5% and 0.3‰ (RSD, *n* = 3) for total N concentration and δ^15^N, respectively. Reference samples of atropine, cellulose, and NIST 1515 apple leaves were used to calibrate the total N concentration measurements (∼2% RSD), while IAEA-600, IAEA-N-2, USGS40, and USGS41 were used to calibrate the N isotope measurements (∼0.15‰ RSD).

### 2.3 Adjacent Peat and Lake Sediment Archives

Our earlier work at the Vindeln Experimental Forest reported Hg concentrations in peat profiles near the larch forest stand,^45^ and Hg deposition flux was calculated to reconstruct the trend of the site’s atmospheric Hg(0) history. In addition, varved sediment Hg/C ratio of the background lake Nylandssjön (64°14′N, 19°46′E, Figure 1a),^46^ located 150 km south of the tree-ring sampling site, was adopted to characterize the regional history of atmospheric Hg(0) concentration.

### 2.4 Atmospheric Hg(0) Measurements

Atmospheric Hg(0) concentrations at Svartberget (Figure 1a) were measured at high frequency (5 min) using an automated continuous Mercury vapor analyzer (Tekran 2537A, Tekran Inc., Canada) and reported as 1 h averages for the year 2018. The sampling inlet was placed at a height of 32.5 m on the Svartberget master tower.^36^ The Tekran 2537A was calibrated automatically at an interval of 47 h using the internal Hg(0) permeation source. The instrument was manually calibrated every 4–6 months using a Tekran 2505 (Tekran Inc., Canada) Hg vapor generator. Atmospheric Hg(0) concentrations at the Mace Head station, western Europe, were measured with a similar method and reported in previous studies.^47,48^ Atmospheric Hg(0) at Pallas and Bredkälen (Northern Europe), that have been reported earlier,^49,50^ were collected by semiautomatic sampling on gold traps at an air sampling rate of 300 mL min^–1^ during 24 h periods, 2 days per week. The samples were later analyzed by using a cold vapor atomic fluorescence spectrometer (Tekran 2500, Tekran Inc., Canada) at the Swedish Environmental Research Institute in Gothenburg. The annual means for atmospheric Hg(0) data across Europe were obtained from the European Monitoring and Evaluation Program (EMEP) as reported in earlier publications.^51,52^

## 3 Results and Discussion

### 3.1 Contrasting Hg Records in Collocated Coniferous Tree Rings

**Figure 2:**
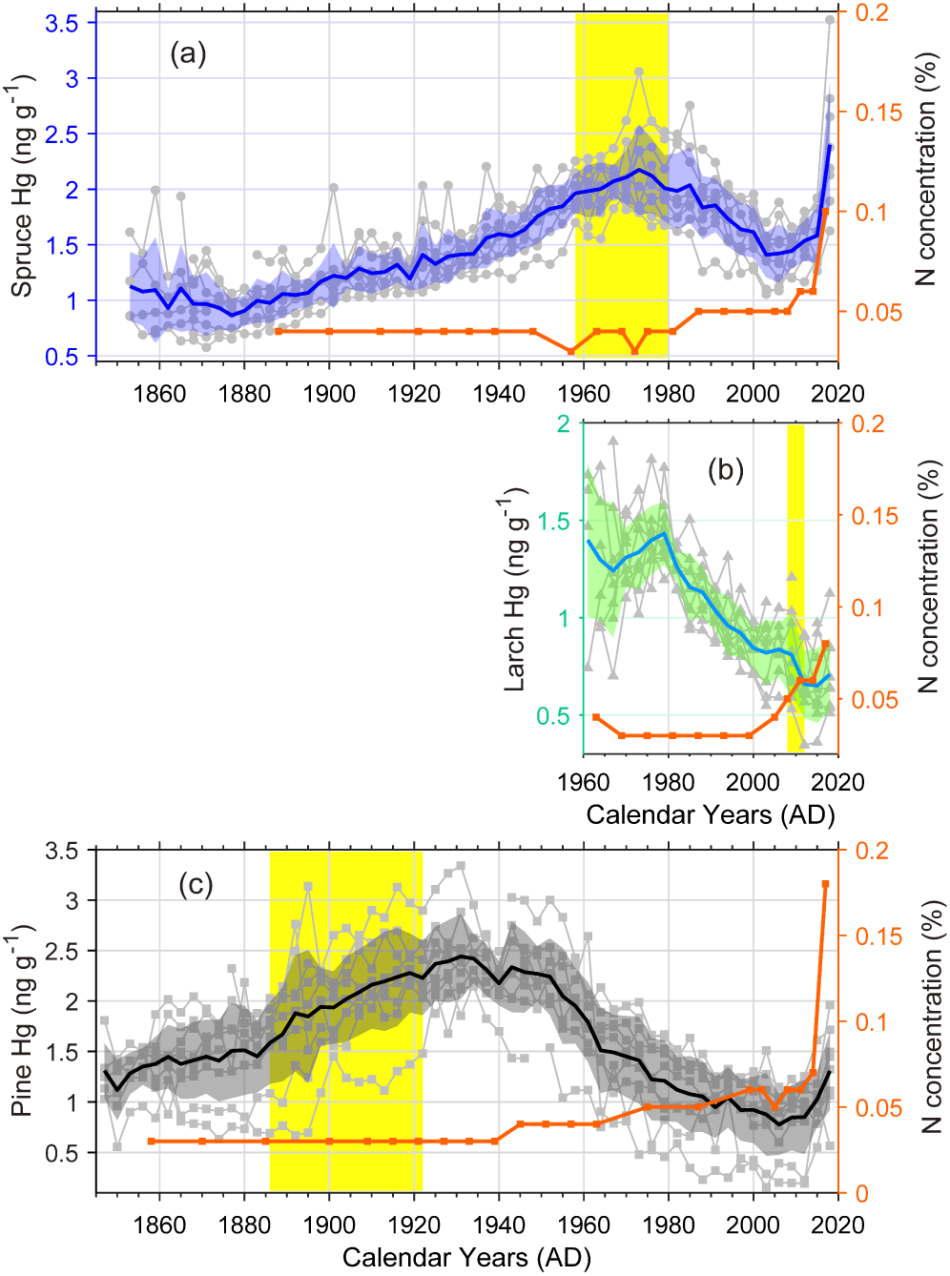
Total Hg and N concentrations recorded in (a) Norway spruce, (b) European larch, and (c) Scots pine tree rings at Svartberget, northern Sweden. Gray circles, triangles, and squares denote three-year segment tree rings Hg concentrations in each individual tree. Shaded areas represent standard deviation with solid lines denoting overall mean values. The yellow bands represent the heartwood-sapwood boundaries.

Tree rings from Norway spruce, Scots pine, and European larch were dated to cover the growth period of 1853–2020, 1847–2020, and 1961–2020, respectively. Correspondingly, the overall mean (±SD) concentrations of total Hg were 1.47 ± 0.40, 1.62 ± 0.51, and 1.06 ± 0.27 ng g^–1^, with a range of 0.86 to 2.40, 0.78 to 2.44, and 0.65 to 1.43 ng g^–1^, respectively (Figure 2). The tree-ring Hg concentration in Scots pine was significantly higher (*p* < 0.01, ANOVA, Figure S1) than in Norway spruce (∼10% higher) and European larch (∼56% higher). Higher Hg concentrations in pine than in spruce or larch have also been observed in previous studies.^23,27,35,53^ The slightly lower tree-ring Hg concentration in spruce compared to pine may be related to the higher canopy gas exchange of pine, which has been observed along a climatic gradient across Europe.^54^ In contrast, the substantially lower tree-ring Hg concentration in larch trees could be attributed to the short period of canopy gas-exchange in this deciduous tree, and therefore less opportunity for Hg(0) uptake than in the evergreen coniferous trees (i.e., pine and spruce trees).^23,30^ Our observed Hg concentration differences between European larch and Scots pine in northern Sweden were similar to the results that were observed at background sites in Central Europe.^55^ Interestingly, N concentrations of tree rings with living cells (e.g., 2018–2020) in Scots pine, Norway spruce, and European larch exhibited decreasing patterns similar to those for Hg concentrations (Figure 2). This suggests that the abundance of nitrogenous solute, controlled by parenchyma cells volume,^56^ may contribute to the differences of Hg concentrations among the three tree species. It should be noted that other species-specific physiological factors (e.g., stomatal conductance, leaf area, and epidermis properties)^2,23^ that impact Hg assimilation by trees may also influence Hg concentrations in tree rings. This, however, requires further investigation. In addition to the tree ring Hg concentration differences between tree species, there was notable variability among individual trees of the same species, especially the Scots pine (Figure 2). The mean individual-tree average tree ring Hg concentrations ranged from 1.44 to 1.63 for Norway spruce, 1.05 to 1.74 for Scots pine, and 0.98 to 1.04 ng g^–1^ for European larch, respectively (Tables S1–S3). Despite variations among species, all members within a species showed consistent patterns. Notable variances of Hg concentrations in tree rings from individual trees of the same species and collocated trees of different species have been previously observed at both background and contaminated sites.^21,26,27,30,32,57^

**Figure 3:**
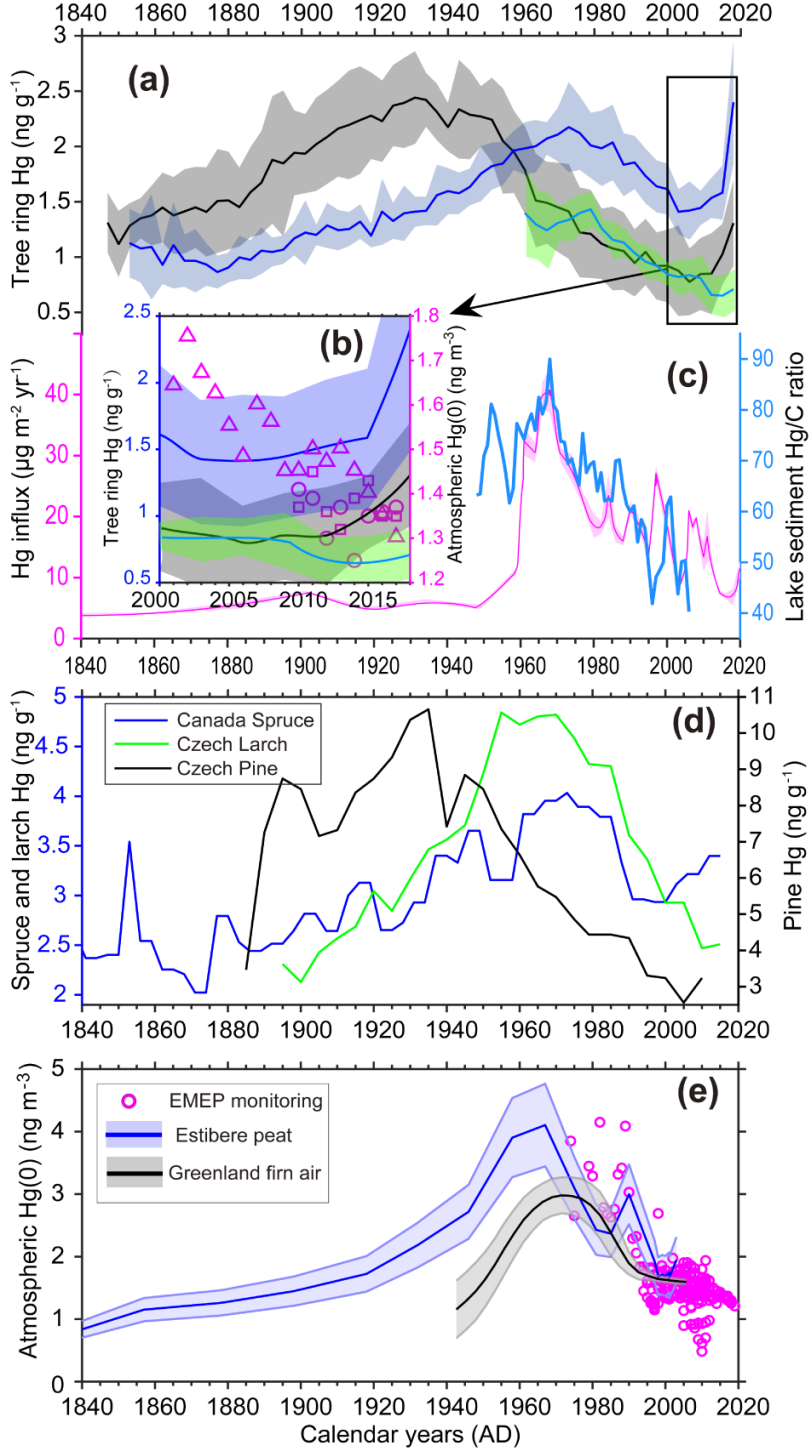
Comparisons of atmospheric Hg concentration measurements and records. (a) Time-series of Hg concentration in northern Sweden tree-ring Hg (black, blue, and cyan-green colors represent pine, spruce, and larch, respectively). (b) Zoomed in tree-rings Hg concentration during the last two decades reconstructed in this study, with atmospheric Hg(0) concentration monitored at Mace Head (triangles),^47,48^ Bredkälen (circles), and Pallas (squares).^50^ (c) Varved lake sediment Hg/C ratio (thick light-blue line) from lake Nylandssjön^46^ and Hg influxes (magenta solid line with the shaded area showing standard deviation) recorded in the Degerö Stormyr peat profile.^45^ (d) Tree-ring Hg profiles from remote sites in Central Europe (Czech Republic)^22,31^ and North America (Canada) reported in earlier publications.^32^ (e) Atmospheric Hg(0) at Northern Hemisphere remote background sites: annual mean atmospheric Hg(0) concentration monitored at EMEP stations (open circles) (http://www.emep.int/), reconstructed atmospheric Hg(0) via Estibere peat in France Pyrenees^14^ and polar firn air in Greenland.^19^

The three tree species displayed different temporal patterns in Hg concentrations. Peaks were observed in 1973 for Norway spruce (2.17 ± 0.33 ng g^–1^, mean ± SD), 1979 for European larch (1.43 ± 0.16 ng g^–1^), and 1931 for Scots pine (2.44 ± 0.42 ng g^–1^) (Figures 2 and S2). We focus first on the period prior to ca. 2005. In Norway spruce, concentrations gradually increased from 1.06 ng g^–1^ in the 1850s to 2.10 ng g^–1^ during the 1970s, and thereafter decreased to 1.41 ng g^–1^ (2003–2005). Similarly, in European larch, we observed a trend in which the concentration decreased from the highest plateau of 1.37 ng g^–1^ in the1970s to the lowest value of 0.65 ng g^–1^ (2015–2018). In contrast, Hg concentrations in Scots pine tree rings increased from 1.28 ng g^–1^ in the 1850s to 2.39 ng g^–1^ and peaked in the 1930s, then decreased to 0.78 ng g^–1^ (2006–2009). Such contrasts in temporal patterns among species in northern Sweden are consistent with previous observations from the same genera at background sites in North America (white spruce, Canada),^32^ and Central Europe (European larch and Scots pine, Czech Republic) (Figure 3d).^31^ Despite substantial differences in the magnitudes of Hg concentrations in tree rings from these various locations (Section 3.3), the trends and peaks of Hg records are consistent and significantly correlated (Figure S3).

Interestingly, all three tree species showed a rebound in Hg concentrations in the most recent decade. The concentrations increased from 1.41 ng g^–1^ (2003) to 2.40 ng g^–1^ (2018) for Norway spruce, from 0.65 ng g^–1^ (2015) to 0.71 ng g^–1^ (2018) for European larch, and from 0.78 ng g^–1^ (2006) to 1.31 ng g^–1^ (2018) for Scots pine. Rebound trends of Hg concentrations in the tree rings of the most recent decade have previously been observed in diverse tree species at both contaminated^26−28,58^ and background sites.^21,30−32,53^

### 3.2 Effect of Atmospheric Hg(0) Concentrations on Tree-Rings Hg Record

The reconstruction of atmospheric Hg(0) concentrations at remote background sites in the Northern Hemisphere, using peat archives from the Pyrenees (France)^14^ and polar firn air from Summit (Greenland),^19^ suggests a steady increase from approximately 1.0 ng m^–3^ in the 1800s to 3.5 ng m^–3^ during the 1970s, followed by a decline to 1.7 ng m^–3^ in the 2000s. In addition, global atmospheric Hg(0) monitoring at regional background and remote sites since 1974 documented the highest level of 3.3 ng m^–3^ in the 1970s, followed by a gradual decline to 1.5 ng m^–3^ in the 2000s (Figure 3e). The peat Hg accumulation rate and sediment Hg/C ratio are proxies used in reconstructing historic atmospheric Hg(0) concentrations.^14,46^ The peat Hg deposition flux at Degerö Stormyr and the varved lake sediment Hg/C ratio at Nylandssjön, both adjacent to our sampling locations, show high consistency with the atmospheric Hg (0) reconstruction from the global background sites in the Pyrenees (France) and Summit (Greenland) over the last two centuries (Figure 3c). This suggests that the atmospheric Hg (0) concentrations at our tree ring sampling location share the same general trends as those at remote sites in the Northern Hemisphere from the 1800s to the 2000s. Therefore, Norway spruce and European larch tree ring Hg concentrations resemble the overall trend of atmospheric Hg(0) concentrations from the 1840s to the 2000s. In contrast, Scots pine failed to record the past atmospheric Hg concentrations, with an apparent peak of Hg(0) concentrations in the 1930s rather than the 1970s (Figure 3). It should, however, be noted that peat deposits and lake sediments not only recorded the dry deposition of atmospheric Hg(0) but also the wet deposition of Hg(II).^16^ Extra caution is needed when comparing tree-ring records with these archives since the former mainly reflect changes in atmospheric Hg(0).

Globally, the Hg inventory documented an anthropogenic Hg release peak in the 1890s,^59^ which was rarely recorded in tree rings and sedimentary records at remote sites. This could be attributed to Hg releases during this period originating mainly from Hg and Silver mining in North America and Europe.^60^ These sectors released mostly Hg contaminated solid or liquid wastes, which were emitted into water and deposited in sediments. A limited fraction of the mixture was emitted to the atmosphere. In contrast, the Hg emissions during the 1970s mainly originated from coal combustion, metal smelting, and chlor-alkali production,^59^ which emits volatile Hg(0) directly to the atmosphere and subsequently leads to rapid increases of atmospheric Hg(0). Since these volatile Hg(0) emissions were associated with regional industrial development, there was spatial and temporal variation in the atmospheric Hg(0) concentrations of different regions.^60^ For example, the Schrenk spruce^58^ and the Dahurian larch^53^ tree-ring records from northwest China and northeast China both showed an Hg concentration peak in the 1990s rather than the 1970s. These Asian tree-ring Hg records agree with the ice cores from the Central Tibetan Plateau^17^ and Central Asia,^18^ and also lake sediments in northeast China,^12^ suggesting there were differences in historical atmospheric Hg(0) concentration trends in Asia, Europe, and North America, which were well preserved in spruce and larch tree rings. Taken together, these results suggest that the Hg in tree rings of spruce and larch effectively recorded the past local and regional atmospheric Hg pollution at different locations across the globe until the 2000s.

Alongside the 1960s–1970s peak in European atmospheric Hg concentration recorded by the larch and spruce tree rings at our remote site, our records also show an increase in pine and spruce tree-ring Hg concentrations since the beginning of the 2000s, with a smaller but noticeable rise in larch since the 2010s (Figure 2). On the contrary, the continuous atmospheric Hg(0) monitoring at Mace Head,^48^ Bredkälen, and Pallas—remote background sites in Western and Northern Europe—shows a slight, generally decreasing trend over the last two decades, from ∼1.8 ng m^–3^ in 2000, to ∼1.5 ng m^–3^ in 2010 and ∼1.3 ng^–3^ in 2018 (Figure 3b). Our intermittent atmospheric Hg(0) measurements in the Svartberget area also show this decline.^61,62^ The atmospheric Hg(0) concentrations were 1.7 ± 0.11 ng m^–3^ in August 2009, 1.30 ± 0.15 ng m^–3^ in July 2014, and 1.21 ± 0.10 ng m^–3^ in July 2018 (Figure 1b). This post-2000 declining trend of atmospheric Hg(0) concentration at our site aligns with synthesized monitoring data from European Monitoring and Evaluation Program (EMEP) stations (Figure 3e). The opposing post-2000 trend in tree rings suggests some consistent decoupling mechanism between recent tree rings and the atmosphere.

When aligning the tree-ring Hg concentrations with the atmospheric Hg concentrations prior to the 2000s, excellent linear correlations between the tree ring Hg concentrations and atmospheric Hg(0) concentrations were obtained for Norway spruce (R^2^ = 0.81, *p* < 0.01) and European larch (R^2^ = 0.92, *p* < 0.01). However, an insignificant correlation was found for the Scots pine tree (R^2^ = 0.04, *p* = 0.32) (Figure S4). Taken together with the opposing trends of tree ring Hg concentrations and the atmospheric Hg concentrations during the post-2000s period for all three tree species (Figure 3b), our analysis suggests Norway spruce and European larch are robust archives for atmospheric Hg(0) only until the 2000s but not for the most recent two decades. Scots pine is simply not useful for atmospheric Hg reconstruction.

### 3.3 Effects of Tree Physiology on Hg Record in Tree Rings

Previous studies have documented an ambiguous rebound peak in tree-ring Hg concentration in the recent decade using low resolution (5–10 years segment) measurements.^21,27,28,31,32,53^ Mechanistic controls for this, however, remain largely unknown. Recently, Mclagan et al., (2022),^27^ observed elevated Hg concentrations in the most recent ten years of tree rings in Norway spruce and European larch from both a HgCl_2_-contaminated site and a nearby uncontaminated site. They attributed this to Hg retention in the xylem solution or the structure/chemistry of the sapwood, although the sapwood length was not identified and the elevated Hg concentrations in the tree rings did not span the entire sapwood.

In our study, we observed a synchronized increase in Hg and N concentrations over the past decade in all three tree species, although atmospheric Hg(0) concentrations have fallen (Figure 2). The N concentrations remained stable at base level from 1840 to 2010, ranging from 0.03 to 0.06%, with mean values of 0.043% in Norway spruce, 0.040% in Scots pine, and 0.037% in European larch. However, N concentrations rose by ∼2–3 times in the last 3 segments of tree rings (with most of the increase emerging in the last segment). At the same time, a 10–40% increase in Hg concentration was observed. This could be attributed to the fact that the outer rings contain large proportions of living parenchyma cells, which have a high concentration of total N and Hg.^35,63^ Xylem parenchyma cells store and reallocate N in the form of amino acids, proteins, and other nitrogenous compounds. Their metabolic activities control the N concentrations in tree rings.^64^ In addition, the concentration gradient between the living phloem and nearby sapwood could cause phloem solute, potentially containing nutrients (e.g., protein and amino acids) and metals (e.g., Hg and Pb), to radially permeate into the inner xylem.^65^ It should be noted that the potential contamination of tree rings during the sampling process was minimized because the bark and living phloem were removed before coring.

The primary pathway for plant assimilation of Hg is foliar uptake of atmospheric Hg(0) via stomata,^18,33,63^ followed by oxidation to Hg(II) and formation of Hg(II)-thiol and HgS species.^66^ However, the potential for subsequent processes to translocate Hg and fix it into the xylem remain open questions. There are three major pathways that regulate the movement of water, minerals, and nutrients in trees: (1) sap water and minerals ascend via the xylem, (2) nutritive sap descends via the phloem, and (3) radial transport of sap occurs via ray cells and bordered pits.^67,68^ As phloem flow is the only downward pathway in the tree, Hg(II) in foliage is likely translocated downward through the phloem along with photosynthates in the aqueous apoplast to sink tissues, such as the tree vessels and xylem.

During the downward phloem transport of photosynthates, including sugar and other organic compounds (e.g., cysteine and other proteinogenic amino acids), Hg(II) may accidentally be cotransported downward through binding to organic compounds that contain reduced sulfur groups. Hg(II) is then expected to be fixed along with the unloading of nutrients to the xylem parenchyma cells.^68^ Evidence of this downward phloem transport of Hg and fixation in the newly formed xylem cells is provided by declining Hg concentrations in tree rings as tree height decreases.^69^ Later, as the tree ages, wood cells die and tissues senescence. During this process, nitrogenous solutes (including associated Hg and other metals) are translocated radially in the xylem rings from the old wood cells to the active young wood cells,^70−73^ resulting in consistently lower N concentrations in the inactive heartwood and less active inner sapwood cells (Figure 2). On the contrary, the active wood cells can receive both the outward-translocated and downward-transported nutrients, where the majority of Hg(II) was bound due to their high thiol content, resulting in the mobilization of Hg in trees. This explains why both N and Hg are anomalously high in the outermost rings, and could also be the reason for the high Hg concentration in the living phloem.^35,74^ This interpretation is supported by the relatively consistent δ^15^N values observed in the heartwood tree rings, alongside a noticeable declining trend of δ^15^N values from the older to the younger rings after the sapwood-heartwood boundary (Figure S5).

Tree physiological processes such as sapwood senescence, heartwood formation, and parenchyma cell death influence wood cell activities, causing chemical elements to migrate both outward and inward.^23,24,75^ Therefore, the formation and location of the sapwood–heartwood boundary are believed to play an important role in the radial translocation of trace elements, including Hg, in tree rings.^30,35,53^ Our results show that the heartwood–sapwood boundary of tree rings is the oldest and most variable in Scots pine (1986–1922), followed by Norway spruce (1958–1980), and is least old in European larch (2008–2012) (Figure 2). Similar observations of much older sapwood in Scots pine than European larch trees was found in Central Europe as indicated by substantially higher water content in sapwood.^35^ The prolonged survival of sapwood in Scots pine trees suggests that the outer 100–140 years of tree rings may retain living parenchyma cells and thus pathways for solute transport.^35,76^ This implies that the radial translocation of peak Hg concentration from the 1970s to the 1930s in Scots pine tree rings could be linked to the migration of xylem sap water or nutrients. However, further investigation of these mechanisms is needed. Nevertheless, the younger and narrower sapwood–heartwood boundary in European larch and Norway spruce supports the notion that the biologically inactive tree ring cells of these species allow them to serve as a robust archive for atmospheric Hg.

For each tree genus from Northern Hemisphere background sites, the overall trends of tree ring Hg concentrations were comparable (Figure 3a vs d, Figure S3). The consistent temporal trends of Hg concentrations in spruce and larch tree rings across the Northern Hemisphere background sites highlight their applicability as a robust atmospheric archive. However, pine trees consistently failed to record the chronology of past atmospheric Hg levels. The absolute magnitudes of Hg concentrations in tree rings showed substantial differences, with the highest levels observed in Central Europe, followed by Canada, and the lowest levels observed in northern Sweden. The peak Hg concentrations in tree rings of Central European Scots pine (7.9 ng g^–1^) and European larch (4.8 ng g^–1^) are 3.7 and 3.4 times higher, respectively, than those in northern Sweden.^35^ We suggest the substantial systematic differences in absolute Hg concentrations between geographic locations may partly be attributed to environmental factors.^23^ The atmospheric Hg(0) concentration in northern Sweden ranged in 1.3–1.7 ng m^–3^ during 2009–2018 (Figure 1b), while it was ∼1.6 ng m^–3^ during the period of 2011 to 2015 at background sites in Central Europe.^31^ The comparable atmospheric Hg(0) concentrations at the two regions do not support the 3–4 times difference in pine and larch tree ring Hg concentrations. It has been noted environmental and growing conditions could influence the assimilation of trace elements into the xylem and, consequently, their concentrations in tree rings.^77^ We propose that environmental and biological factors influencing the growing period (i.e., canopy gas-exchange time) and photosynthesis (e.g., stomatal conductance, temperature) during tree ring formation may have a significant impact on net Hg assimilation by trees, similar to that of photosynthates. The length of growing time was found to be linearly correlated with foliar Hg concentrations,^78^ and thus may exert a major control on tree ring Hg concentrations. It has also been demonstrated that photosynthetic capacity is positively correlated with foliar Hg uptake rates,^78,79^ which might be proportionate to the Hg concentrations in tree rings. The growing season in Central Europe, characterized by a mean annual air temperature of 7.9 °C and total precipitation of 687 mm, is much longer and warmer than in northern Sweden (2.1 and 619 mm, respectively) (Figure 1c), leading to a longer period of canopy gas-exchange and greater net photosynthesis in Central Europe. The synergy effects of the above two factors may promote Hg assimilation by trees and result in higher Hg concentrations in Central Europe (Figure 3). Environmental change, such as climate change, influences the growing period and photosynthesis of trees. Thus, environmental change may also impact Hg concentrations in tree rings, and this warrants further investigation.

## 4 Implications

Our study demonstrates that Hg concentrations recorded in tree rings are jointly controlled by the atmospheric Hg concentration and species-dependent physiological characteristics of the trees. The Hg records in tree rings likely involve two dynamic steps. First, during tree growth, atmospheric Hg assimilated by foliage is translocated to newly formed wood cells along with nutritive sap descending via the phloem. Second, a substantial fraction of Hg may translocate radially with nitrogenous solutes from aging wood cells to younger, active cells during wood cell death and tissue senescence, resulting in stable Hg records only in biologically inactive heartwood cells. These mechanistic insights into Hg assimilation and stabilization in tree rings have important implications for using dendrochemistry to reconstruct past atmospheric Hg(0) concentrations. From these insights, we can better understand why Norway spruce (*Picea abies*) and European larch (*Larix decidua*) are robust archives for atmospheric Hg(0) reconstruction from the 1840s (when the trees started growing) to the 2000s but not for the recent decade. The physiology of Scots pine (*Pinus sylvestris*) also explains why it is not suitable for past atmospheric Hg reconstruction. We suggest that future studies carefully consider tree physiology when using dendrochemistry as an atmospheric Hg archive by applying the following three criteria: (1) Select suitable tree species with narrow active sapwood^35^ and an unambiguous heartwood–sapwood boundary, such as European larch and Norway spruce. (2) Use only tree rings with biologically inactive wood cells (i.e., heartwood and inner inactive sapwood tree rings) to reliably reconstruct the past atmospheric Hg. This can be assessed by measuring wood cell activities with biological indicators such as nitrogen concentrations. (3) Consider geographical-location-related environmental factors such as temperature and annual tree growth period, which influence net Hg assimilation by tree rings and cause systematic differences in the absolute magnitude of Hg concentrations recorded in tree rings, although not in the overall trend. In addition, to protect the trees from fungal and bacterial infections, we recommend future studies to use gardening wax to seal residual holes after retrieval of the tree-ring cores.

Reconstructing decadal to centennial atmospheric Hg(0) concentrations at local, regional, and global scales is essential for assessing the environmental effectiveness of the Minamata Convention. Tree dendrochemistry, a cost-efficient, high-resolution, and easily accessible natural archive, can serve as a reliable proxy for atmospheric Hg. Our study highlights the role of tree physiology in the assimilation and translocation of Hg in tree rings. At the very least, using N enrichment to filter out the rings with high physiological activity is necessary to avoid physiological interference with predictions of atmospheric Hg. This filtering will increase the effectiveness of attempts to use tree rings for reconstructing local to regional atmospheric Hg concentrations that are essential for assessing the effectiveness of Hg control efforts related to the Minamata Convention. Future studies advancing molecular insights into Hg assimilation, mobilization, and stabilization in wood cells during the formation, growth, and aging of tree rings will further enhance the development of tree dendrochemistry as an effective atmospheric Hg archive.

## Supporting Information Available

Detailed information on total Hg concentrations in all tree ring samples, trend analysis of mean Hg concentrations recorded in tree rings, total N concentrations and δ^15^N values in tree rings, and ancillary data analysis (PDF)
