# Supplementary material for: Tree Rings Mercury Controlled by Atmospheric Gaseous Elemental Mercury and Tree Physiology
Source: Environ Sci Technol. 2024 Sep 9;58(38):16833–42. doi: 10.1021/acs.est.4c05662 (PMC11428168; doi:10.1021/acs.est.4c05662)
Supplement: Supplementary file 1 — es4c05662_si_001.pdf [file es4c05662_si_001.pdf]

Supporting Information

Tree Rings Mercury Controlled by Atmospheric Gaseous Elemental Mercury and Tree Physiology

Haijun Peng [1], Xiangwen Zhang [1,2], Kevin Bishop [3], John Marshall [1], Mats B. Nilsson [1], Chuxian Li [1,4], Erik Björn [5], Wei Zhu [1,*]

[1] Department of Forest Ecology and Management, Swedish University of Agricultural Sciences, SE-90183 Umeå, Sweden

[2] School of Resources & Environment, Nanchang University, Nanchang 330031, China

[3] Department of Aquatic Sciences and Assessment, Swedish University of Agricultural Sciences, SE-75651 Uppsala, Sweden

[4] Institute of Geography and Oeschger Center for Climate Change Research, University of Bern, 3012 Bern, Switzerland

[5] Department of Chemistry, Umeå University, SE-901 87 Umeå, Sweden

***** Corresponding Author:***

Wei Zhu, Phone: +46 (0)76 9682807; E-mail: wei.zhu@slu.se; zhuweicas@gmail.com

SI list of contents: 13 pages, 3 tables, and 5 figures.

Tables: Table S1 – Table S3 (Pages 2 – 8)
Figures: Figure S1 – Figure S5 (Pages 9 – 13)
References: (Page 13)

Table S1. Total Hg concentration in tree rings of 9 Norway spruce trees. Note: NaN represents a missing sample caused by faults during sample processing and analysis or by data beyond the starting year of tree growth.

Year (AD)	Spruce 1 (μg kg^{-1})	Spruce 2 (μg kg^{-1})	Spruce 3 (μg kg^{-1})	Spruce 4 (μg kg^{-1})	Spruce 5 (μg kg^{-1})	Spruce 6 (μg kg^{-1})	Spruce 7 (μg kg^{-1})	Spruce 8 (μg kg^{-1})	Spruce 9 (μg kg^{-1})	Mean (μg kg^{-1})	Standard deviation (μg kg^{-1})
2018	NaN	2.82	3.52	2.38	2.13	1.89	2.19	1.62	2.65	2.40	0.56
2015	1.83	1.33	1.92	1.36	1.92	1.62	1.62	1.26	1.38	1.58	0.25
2012	1.80	1.39	1.72	1.63	1.50	1.46	1.67	1.49	1.17	1.54	0.18
2009	1.48	1.20	1.33	1.56	1.46	1.57	1.66	1.53	1.20	1.44	0.16
2006	1.51	1.36	1.09	1.33	1.77	1.54	1.88	1.12	1.19	1.42	0.26
2003	1.51	1.58	1.10	1.38	1.46	1.56	1.70	1.05	1.35	1.41	0.20
2000	1.95	1.53	1.30	1.88	1.74	1.57	1.79	1.28	1.47	1.61	0.23
1997	1.91	1.77	1.24	1.90	1.48	1.61	1.93	1.45	1.46	1.64	0.23
1994	2.01	2.04	1.73	1.80	1.56	1.75	1.94	1.23	1.53	1.73	0.25
1991	1.88	1.93	1.81	2.24	1.96	1.74	2.09	1.35	1.69	1.86	0.24
1988	1.73	1.93	1.81	2.36	1.81	1.98	2.01	1.26	1.62	1.83	0.28
1985	1.73	2.75	1.95	2.38	1.96	1.86	2.24	1.60	1.85	2.04	0.34
1982	1.62	2.44	1.74	2.50	1.91	1.87	2.41	1.62	1.75	1.98	0.34
1979	1.72	2.45	1.91	2.52	1.96	1.95	2.18	1.67	1.72	2.01	0.30
1976	1.66	2.61	2.21	2.61	2.04	1.89	2.38	1.75	1.93	2.12	0.33
1973	1.79	3.06	1.85	2.53	2.35	2.02	2.34	1.84	1.80	2.17	0.41
1970	1.82	2.62	1.90	2.07	2.05	2.09	1.96	2.43	2.02	2.11	0.24
1967	2.00	2.37	2.03	2.15	2.11	2.04	1.82	1.94	2.18	2.07	0.15
1964	1.83	2.29	2.03	2.22	2.19	1.90	1.55	2.21	1.79	2.00	0.24
1961	2.27	2.28	1.66	2.07	2.19	2.03	1.58	1.86	1.92	1.98	0.24

Continue to the next page

Table S1 (Continued)

Year (AD)	Spruce 1 (μg kg^{-1})	Spruce 2 (μg kg^{-1})	Spruce 3 (μg kg^{-1})	Spruce 4 (μg kg^{-1})	Spruce 5 (μg kg^{-1})	Spruce 6 (μg kg^{-1})	Spruce 7 (μg kg^{-1})	Spruce 8 (μg kg^{-1})	Spruce 9 (μg kg^{-1})	Mean (μg kg^{-1})	Standard deviation (μg kg^{-1})
1958	1.87	2.25	2.03	2.15	1.88	1.82	1.70	2.14	1.84	1.96	0.18
1955	1.83	1.95	1.82	2.19	1.99	1.83	1.44	1.88	1.69	1.85	0.20
1952	1.80	1.86	1.97	2.00	1.98	1.62	1.32	2.00	1.85	1.82	0.21
1949	1.71	1.72	1.49	1.96	1.81	1.52	1.38	2.19	1.99	1.75	0.25
1946	1.64	1.81	1.61	1.55	1.77	1.45	1.31	1.83	1.74	1.63	0.17
1943	1.68	1.58	1.55	1.43	1.81	1.51	1.19	1.85	1.59	1.58	0.19
1940	1.58	1.39	1.66	1.39	1.93	1.84	1.25	1.69	1.63	1.60	0.21
1937	1.38	1.36	1.65	1.50	1.76	2.20	1.23	1.56	1.38	1.56	0.27
1934	1.39	1.55	1.36	1.46	1.64	1.48	1.20	1.41	1.28	1.42	0.13
1931	1.28	1.44	1.60	1.56	1.55	1.34	1.24	1.46	1.23	1.41	0.14
1928	1.22	1.40	1.55	1.23	1.40	1.28	2.03	1.21	1.24	1.40	0.25
1925	1.13	1.29	1.68	1.37	1.43	1.43	1.18	1.26	1.18	1.33	0.16
1922	1.09	1.34	1.72	1.14	1.48	1.30	1.53	2.03	1.07	1.41	0.30
1919	1.10	1.09	1.37	1.33	1.24	1.23	1.23	1.09	1.07	1.19	0.11
1916	1.43	1.13	1.58	1.32	1.39	1.37	1.44	1.17	1.06	1.32	0.16
1913	1.17	1.06	1.33	1.06	1.29	1.39	1.49	1.09	1.40	1.25	0.15
1910	1.26	1.21	1.15	1.06	1.07	1.39	1.41	1.03	1.61	1.24	0.18
1907	1.25	1.03	1.28	1.12	1.21	1.61	1.58	1.04	1.44	1.29	0.20
1904	1.20	1.02	1.31	1.09	1.21	1.34	1.20	1.20	1.23	1.20	0.09
1901	1.17	0.99	1.05	1.04	1.10	2.01	1.16	1.28	1.19	1.22	0.29
1898	1.18	0.88	1.20	0.99	1.23	1.47	1.19	1.21	1.17	1.17	0.15
1895	1.11	0.88	1.09	0.94	1.03	1.34	1.08	0.97	1.16	1.07	0.13

Continue to the next page

Table S1 (Continued)

Year (AD)	Spruce 1 (μg kg^{-1})	Spruce 2 (μg kg^{-1})	Spruce 3 (μg kg^{-1})	Spruce 4 (μg kg^{-1})	Spruce 5 (μg kg^{-1})	Spruce 6 (μg kg^{-1})	Spruce 7 (μg kg^{-1})	Spruce 8 (μg kg^{-1})	Spruce 9 (μg kg^{-1})	Mean (μg kg^{-1})	Standard deviation (μg kg^{-1})
1892	1.17	0.79	1.15	0.88	1.01	1.21	1.06	1.05	1.09	1.05	0.13
1892	1.17	0.79	1.15	0.88	1.01	1.21	1.06	1.05	1.09	1.05	0.13
1889	1.09	0.76	1.35	0.88	0.91	1.46	1.03	1.00	1.04	1.06	0.21
1886	1.00	0.72	0.80	0.92	0.84	1.35	1.04	1.11	1.01	0.97	0.18
1883	1.07	1.04	0.69	0.79	0.84	1.41	0.97	1.10	1.06	1.00	0.20
1880	0.98	0.82	0.70	0.84	0.83	NaN	1.10	0.87	1.11	0.91	0.13
1877	1.00	0.66	0.75	0.65	0.94	NaN	0.97	0.88	1.06	0.86	0.15
1874	0.95	0.72	0.73	0.66	1.19	NaN	1.02	1.04	1.16	0.93	0.19
1871	0.96	0.67	0.78	0.58	1.45	NaN	1.07	0.90	1.33	0.97	0.29
1868	0.97	0.70	0.82	0.64	1.19	NaN	1.37	0.90	1.15	0.97	0.24
1865	0.91	1.94	0.79	0.63	1.12	NaN	NaN	1.25	1.11	1.11	0.39
1862	0.88	NaN	0.77	0.76	NaN	NaN	NaN	1.22	1.03	0.93	0.17
1859	0.89	NaN	0.71	0.73	NaN	NaN	NaN	1.99	1.14	1.09	0.47
1856	0.87	NaN	0.69	NaN	NaN	NaN	NaN	1.42	1.33	1.08	0.31
1853	0.86	NaN	0.86	NaN	NaN	NaN	NaN	1.61	1.17	1.13	0.31

Table S2. Total Hg concentration in tree rings of 9 Scots pine trees.

Year (AD)	Pine 1 (µg kg^{-1})	Pine 2 (µg kg^{-1})	Pine 3 (µg kg^{-1})	Pine 4 (µg kg^{-1})	Pine 5 (µg kg^{-1})	Pine 6 (µg kg^{-1})	Pine 7 (µg kg^{-1})	Pine 8 (µg kg^{-1})	Pine 9 (µg kg^{-1})	Mean (µg kg^{-1})	Standard deviation (µg kg^{-1})
2018	1.50	1.96	1.08	1.06	1.11	0.56	1.45	1.32	1.72	1.31	0.39
2015	1.06	1.18	0.90	1.02	0.55	1.06	1.08	0.78	1.61	1.03	0.27
2012	1.12	1.18	1.00	0.90	0.23	0.19	0.81	0.96	1.25	0.85	0.37
2009	1.23	0.94	1.03	1.01	0.36	0.20	0.80	0.95	1.11	0.85	0.32
2006	1.06	0.90	0.89	0.76	0.39	0.20	0.60	1.02	1.15	0.78	0.30
2003	1.51	0.92	0.86	0.98	0.15	0.29	0.78	1.12	1.27	0.88	0.41
2000	1.11	0.96	1.27	0.89	0.36	0.42	0.77	1.20	1.30	0.92	0.33
1997	1.18	0.97	0.93	0.96	0.44	0.34	1.01	1.20	1.24	0.92	0.30
1994	1.27	1.05	1.06	1.15	0.25	1.06	1.02	1.33	1.23	1.05	0.30
1991	1.24	1.04	0.99	1.05	0.32	0.52	0.92	1.17	1.25	0.95	0.30
1988	1.32	1.05	1.35	1.01	0.23	0.98	0.83	1.24	1.48	1.05	0.35
1985	1.32	1.21	1.37	0.94	0.35	0.66	0.91	1.63	1.32	1.08	0.38
1982	1.37	1.25	1.52	0.94	0.59	0.69	0.95	1.42	1.34	1.12	0.32
1979	1.48	1.36	1.64	0.94	0.46	0.82	1.00	1.67	1.53	1.21	0.40
1976	1.52	1.24	1.66	1.16	0.53	0.58	1.13	1.65	1.54	1.22	0.41
1973	1.73	1.48	1.99	1.34	0.48	1.13	1.17	1.83	1.56	1.41	0.43
1970	1.50	1.61	1.95	1.39	0.81	0.87	1.20	1.95	1.78	1.45	0.40
1967	1.64	1.77	1.88	1.23	0.79	1.36	1.09	1.92	1.74	1.49	0.37
1964	1.62	1.75	1.89	1.40	0.90	0.75	1.43	2.14	1.71	1.51	0.42
1961	1.78	1.73	2.07	1.58	2.64	1.12	1.49	1.94	1.75	1.79	0.40
1958	1.88	1.85	2.36	1.71	2.21	1.12	2.03	2.43	1.98	1.95	0.37
1955	2.07	2.45	2.34	1.73	2.04	1.04	1.81	2.83	2.13	2.05	0.48

Continue to the next page

Table S2 (Continued)

Year (AD)	Pine 1 (μg kg^{-1})	Pine 2 (μg kg^{-1})	Pine 3 (μg kg^{-1})	Pine 4 (μg kg^{-1})	Pine 5 (μg kg^{-1})	Pine 6 (μg kg^{-1})	Pine 7 (μg kg^{-1})	Pine 8 (μg kg^{-1})	Pine 9 (μg kg^{-1})	Mean (μg kg^{-1})	Standard deviation (μg kg^{-1})
1952	2.23	2.27	2.39	2.04	1.97	1.54	2.53	3.00	2.20	2.24	0.38
1949	2.15	1.97	2.19	1.92	2.46	NaN	2.50	2.79	2.17	2.27	0.27
1946	2.16	2.24	2.52	2.00	2.50	1.43	2.40	3.00	2.32	2.29	0.40
1943	2.29	2.02	2.60	2.04	2.59	1.43	2.75	3.03	2.27	2.34	0.45
1940	2.29	2.20	2.24	2.06	2.49	1.70	2.32	NaN	2.11	2.18	0.22
1937	2.19	2.08	2.66	2.44	2.48	1.83	2.20	2.63	2.30	2.31	0.26
1934	2.19	2.30	2.63	2.46	2.95	2.15	2.17	2.46	2.47	2.42	0.24
1931	2.38	2.14	2.45	2.88	3.34	2.12	1.86	2.52	2.28	2.44	0.42
1928	2.28	2.08	2.58	2.50	3.27	1.68	2.21	2.55	2.41	2.39	0.41
1925	2.16	1.88	2.22	2.47	3.11	2.11	2.06	2.74	2.58	2.37	0.37
1922	2.03	2.09	2.14	2.23	2.90	1.48	2.57	2.41	2.20	2.23	0.37
1919	2.16	2.30	2.26	2.23	2.97	1.37	2.43	2.46	2.32	2.28	0.39
1916	2.18	2.22	1.87	2.45	3.13	1.30	2.70	2.30	1.99	2.24	0.49
1913	2.13	2.23	1.84	2.66	2.83	1.11	2.68	2.22	2.05	2.19	0.49
1910	2.20	2.08	2.18	2.32	2.90	1.22	2.58	1.93	2.05	2.16	0.43
1907	2.13	1.90	1.86	2.50	2.57	1.20	2.65	2.04	1.93	2.09	0.42
1904	2.28	1.89	1.55	2.18	2.72	1.24	2.41	1.89	2.02	2.02	0.42
1901	2.07	1.71	1.55	2.18	2.65	1.45	2.12	1.64	2.03	1.94	0.36
1898	2.20	1.74	2.01	2.30	2.30	1.09	2.27	1.59	1.99	1.94	0.38
1895	1.97	1.51	1.77	2.19	2.37	0.70	3.14	1.24	1.74	1.85	0.66
1892	1.90	2.76	1.89	2.07	2.04	0.67	2.50	1.36	1.72	1.88	0.58
1889	1.85	2.05	1.56	2.03	2.04	0.77	2.14	0.99	1.62	1.67	0.47

Continue to the next page

Table S2 (Continued)

Year (AD)	Pine 1 (µg kg^{-1})	Pine 2 (µg kg^{-1})	Pine 3 (µg kg^{-1})	Pine 4 (µg kg^{-1})	Pine 5 (µg kg^{-1})	Pine 6 (µg kg^{-1})	Pine 7 (µg kg^{-1})	Pine 8 (µg kg^{-1})	Pine 9 (µg kg^{-1})	Mean (µg kg^{-1})	Standard deviation (µg kg^{-1})
1886	1.77	1.81	1.76	2.03	1.92	0.69	1.65	1.09	1.56	1.58	0.41
1883	1.70	1.55	1.67	1.76	1.88	0.70	1.38	1.04	1.37	1.45	0.36
1880	1.55	1.68	1.58	1.78	1.88	0.85	2.18	0.73	1.37	1.51	0.44
1877	1.48	1.72	1.71	2.32	2.01	0.94	1.31	0.76	1.31	1.51	0.47
1874	1.52	1.74	1.67	NaN	1.78	0.90	1.73	0.63	1.29	1.41	0.40
1871	1.44	1.88	1.65	1.53	1.92	0.93	1.56	0.77	1.35	1.45	0.37
1868	1.36	1.73	1.45	1.81	2.04	0.94	1.34	0.79	1.26	1.41	0.38
1865	1.49	1.74	1.30	1.60	1.89	0.86	1.46	0.81	1.24	1.38	0.35
1862	1.42	1.87	0.88	1.46	2.01	1.35	1.45	1.21	1.38	1.45	0.32
1859	1.40	1.55	1.14	1.47	1.87	1.23	1.43	0.84	1.46	1.38	0.27
1856	1.34	1.54	NaN	1.24	1.27	1.49	1.50	0.96	1.46	1.35	0.18
1853	1.28	1.29	NaN	1.24	NaN	NaN	1.58	0.90	1.41	1.28	0.20
1850	1.09	1.37	NaN	0.96	NaN	NaN	1.43	0.56	1.31	1.12	0.30
1847	1.03	1.81	NaN	NaN	NaN	NaN	1.33	1.13	1.25	1.31	0.27

Table S3. Total Hg concentration in tree rings of 9 European larch trees.

Year (AD)	Larch 1 (μg kg^{-1})	Larch 2 (μg kg^{-1})	Larch 3 (μg kg^{-1})	Larch 4 (μg kg^{-1})	Larch 5 (μg kg^{-1})	Larch 6 (μg kg^{-1})	Larch 7 (μg kg^{-1})	Larch 8 (μg kg^{-1})	Larch 9 (μg kg^{-1})	Mean (μg kg^{-1})	Standard deviation (μg kg^{-1})
2018	0.85	0.54	0.55	0.69	0.64	0.64	0.84	0.51	1.12	0.71	0.19
2015	0.79	0.55	0.36	0.97	0.57	0.63	0.55	0.51	0.92	0.65	0.19
2012	0.91	0.63	0.35	NaN	0.90	0.60	0.57	0.69	0.62	0.66	0.17
2009	1.03	0.97	0.53	1.21	0.77	0.67	0.68	0.59	0.83	0.81	0.21
2006	1.05	0.82	0.96	NaN	NaN	0.90	0.73	0.59	0.81	0.84	0.14
2003	0.88	0.84	0.67	1.08	0.96	0.87	0.55	0.59	0.94	0.82	0.17
2000	0.88	0.95	0.72	0.83	0.95	0.98	0.71	0.71	0.85	0.84	0.10
1997	1.02	1.04	0.83	0.89	0.93	1.15	0.72	0.92	0.80	0.92	0.12
1994	0.97	0.87	0.80	0.83	1.11	0.95	1.31	0.88	0.90	0.96	0.15
1991	1.14	1.01	1.15	0.98	0.89	1.09	1.15	0.87	1.07	1.04	0.10
1988	1.21	1.23	1.04	0.99	1.26	1.14	1.33	0.94	1.06	1.13	0.13
1985	1.24	1.04	1.30	1.05	1.15	1.24	1.50	0.94	0.96	1.16	0.17
1982	1.24	1.15	1.35	1.24	1.27	1.28	1.38	1.27	1.14	1.26	0.08
1979	1.51	1.31	1.57	1.77	1.20	1.34	1.39	1.46	1.35	1.43	0.16
1976	1.29	1.31	1.81	1.15	1.36	1.45	1.41	1.50	1.31	1.40	0.18
1973	1.02	1.24	1.51	1.26	1.65	1.28	1.46	1.14	1.45	1.34	0.19
1970	1.38	1.19	1.41	1.10	1.53	1.25	1.20	1.17	1.55	1.31	0.15
1967	1.29	1.56	1.23	0.70	1.07	1.17	NaN	1.90	1.00	1.24	0.34
1964	1.11	1.59	1.78	0.97	0.95	1.37	NaN	NaN	NaN	1.30	0.31
1961	0.74	1.73	1.65	NaN	NaN	NaN	NaN	1.47	NaN	1.40	0.39

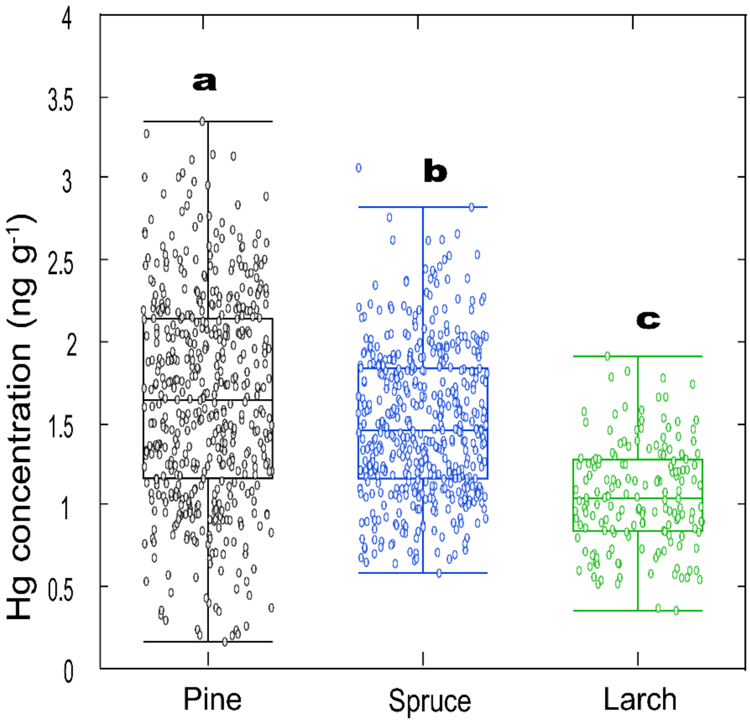

Figure S1. Box plot of Hg concentrations in pine, spruce, and larch tree rings. The box boundaries represent the 25th and 75th percentiles and the solid horizontal line in the box represents the median value. Cross hatches indicate the 5th and 95th percentiles. The circles are the individual Hg concentration measurements. Boxplots designated by the same letter are not significantly different based on the ANOVA with Turkey's HSD post-hoc test ($p < 0.01$).

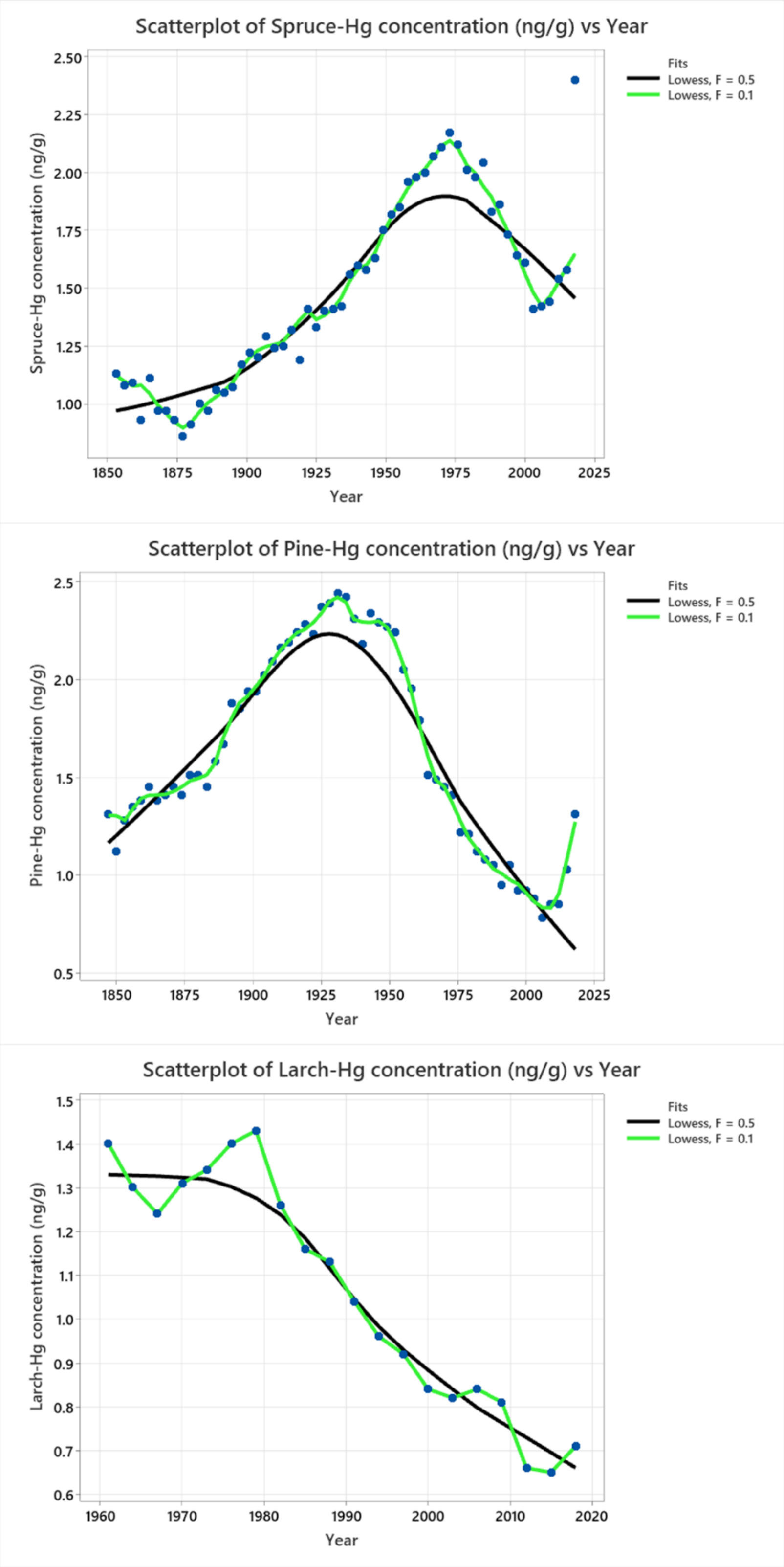

Figure S2. Hg concentrations (mean values) and trends in tree rings from Svartberget (Vindeln Experimental Forest), northern Sweden. Trends analyses were performed with Lowess (locally weighted scatterplot smoothing) under varying degrees of smoothing (df = 0.5 and 0.1).

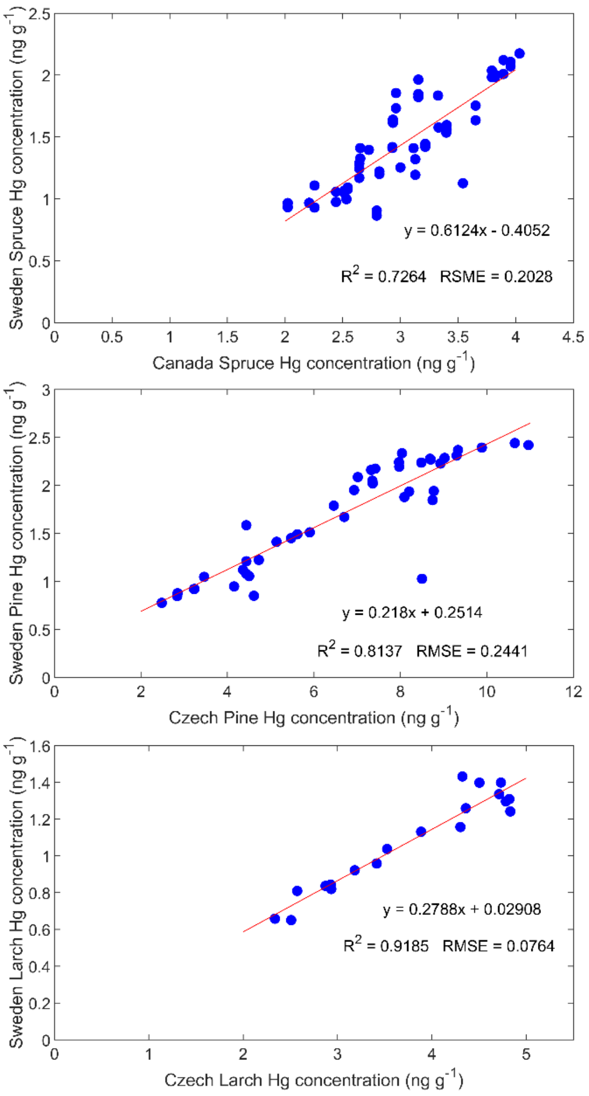

Figure S3. Scatter-plot of tree ring Hg concentrations (spruce, pine and larch trees) from Northern Sweden and from other background sites in North America (Canada) and Central Europe (Czech Republic).[1-3] The data points from Canada and the Czech Republic were interpolated to a 3-year resolution based on a linear spline for aligning the data points into the same years. The red lines in each figure are fitted based on ordinary least squares regression.

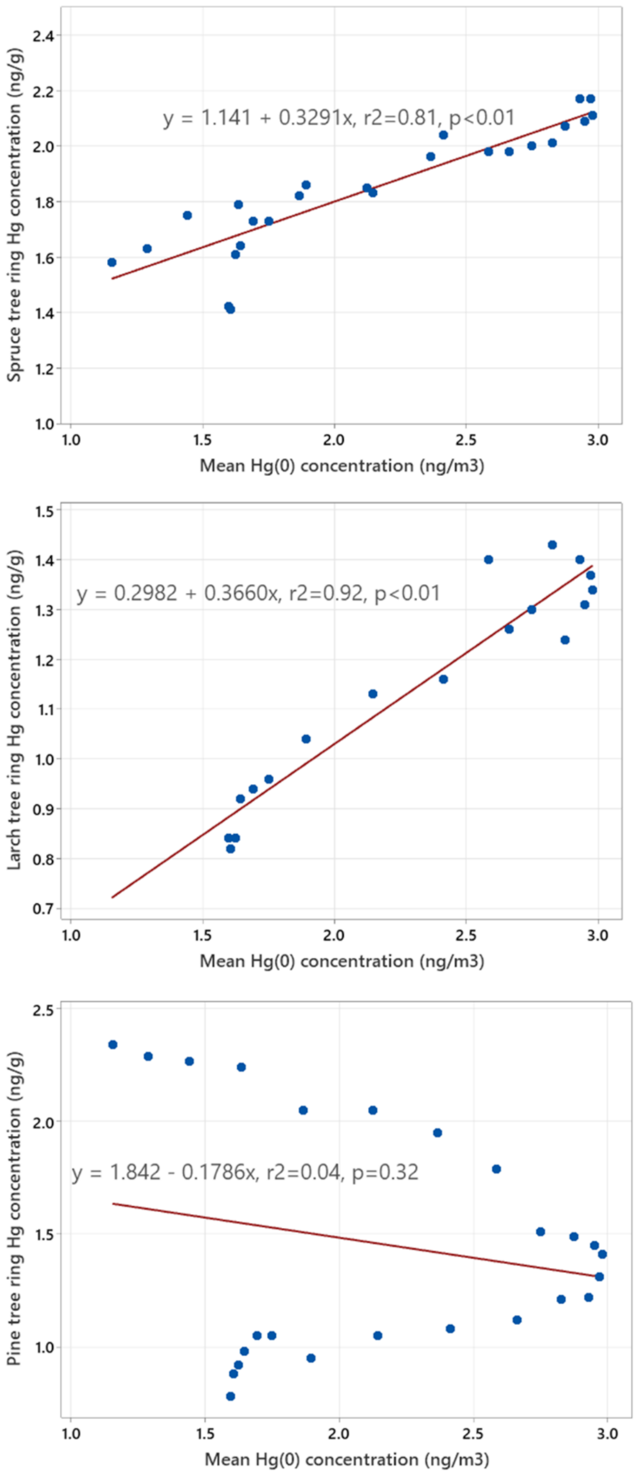

Figure S4. Scatter-plot of tree ring Hg concentrations versus atmospheric Hg(0) concentrations (reconstructed from the polar firn air[4]). The red lines in each figure are fitted based on the ordinary least squares regression.

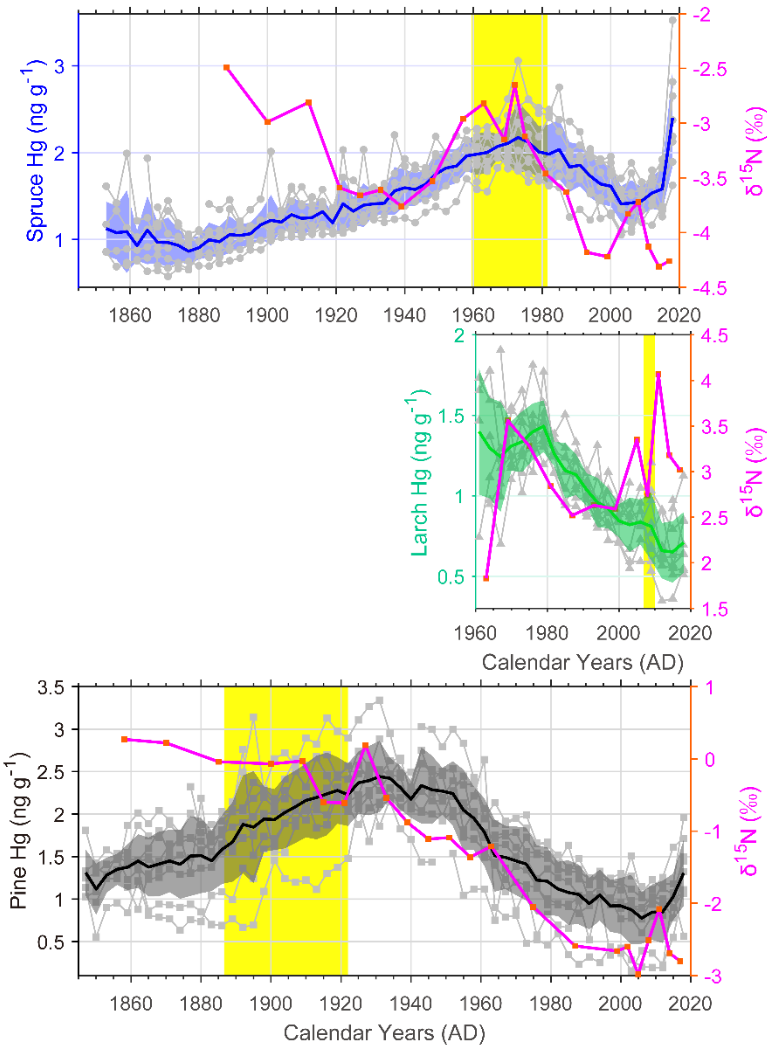

Figure S5. Total Hg concentrations and $\delta^{15}N$ values of total N in tree rings.

References

1. Navrátil, T.; Šimeček, M.; Shanley, J. B.; Rohovec, J.; Hojdová, M.; Houška, J., The history of mercury pollution near the Spolana chlor-alkali plant (Neratovice, Czech Republic) as recorded by Scots pine tree rings and other bioindicators. *Science of The Total Environment* **2017,** *586*, 1182-1192.
2. Navrátil, T.; Nováková, T.; Shanley, J. B.; Rohovec, J.; Matoušková, Š.; Vaňková, M.; Norton, S. A., Larch Tree Rings as a Tool for Reconstructing 20th Century Central European Atmospheric Mercury Trends. *Environ. Sci. Technol.* **2018,** *52*, (19), 11060-11068.
3. Ghotra, A.; Lehnherr, I.; Porter, T. J.; Pisaric, M. F. J., Tree-Ring Inferred Atmospheric Mercury Concentrations in the Mackenzie Delta (NWT, Canada) Peaked in the 1970s but Are Increasing Once More. *ACS Earth and Space Chemistry* **2020,** *4*, (3), 457-466.
4. Faïn, X.; Ferrari, C. P.; Dommergue, A.; Albert, M. R.; Battle, M.; Severinghaus, J.; Arnaud, L.; Barnola, J. M.; Cairns, W.; Barbante, C., Polar firn air reveals large-scale impact of anthropogenic mercury emissions during the 1970s. *Proceedings of the National Academy of Sciences* **2009,** *106*, (38), 16114-16119.
